# Physiological effects of tangeretin and heptamethoxyflavone on obese C57BL/6J mice fed a high‐fat diet and analyses of the metabolites originating from these two polymethoxylated flavones

**DOI:** 10.1002/fsn3.2167

**Published:** 2021-02-12

**Authors:** Marina Nery, Paula S. Ferreira, Danielle R. Gonçalves, Luis C. Spolidorio, John A. Manthey, Thais B. Cesar

**Affiliations:** ^1^ Department of Food and Nutrition Laboratory of Nutrition Faculty of Pharmaceutical Sciences São Paulo State University (UNESP) Araraquara Brazil; ^2^ U.S. Horticultural Research Laboratory Agricultural Research Service USDA Fort Pierce FL USA; ^3^ Department of Physiology and Pathology School of Dentistry São Paulo State University (UNESP) Araraquara Brazil

**Keywords:** citrus, flavonoids, inflammation, metabolites, obesity, oxidative stress

## Abstract

Two compounds from citrus peel, tangeretin (TAN) and 3′,4′,3,5,6,7,8‐heptamethoxyflavone (HMF), were investigated for their abilities to repair metabolic damages caused by an high‐fat diet (HFD) in C57BL/6J mice. In the first 4 weeks, mice were fed either a standard diet (11% kcal from fat) for the control group, or a HFD (45% kcal from fat) to establish obesity in three experimental groups. In the following 4 weeks, two groups receiving the HFD were supplemented with either TAN or HMF at daily doses of 100 mg/kg body weight, while the two remaining groups continued to receive the standard healthy diet or the nonsupplemented HFD. Four weeks of supplementation with TAN and HMF resulted in intermediate levels of blood serum glucose, leptin, resistin, and insulin resistance compared with the healthy control and the nonsupplemented HFD groups. Blood serum peroxidation (TBARS) levels were significantly lower in the TAN and HMF groups compared with the nonsupplemented HFD group. Several differences occurred in the physiological effects of HMF versus TAN. TAN, but not HMF, reduced adipocyte size in the mice with pre‐existent obesity, while HMF, but not TAN, decreased fat accumulation in the liver and also significantly increased the levels of an anti‐inflammatory cytokine, IL‐10. In an analysis of the metabolites of TAN and HMF, several main classes occurred, including a new set of methylglucuronide conjugates. It is suggested that contrasts between the observed physiological effects of TAN and HMF may be attributable to the differences in numbers and chemical structures of TAN and HMF metabolites.

## Graphical abstract

Tangeretin (TAN) and heptamethoxyflavone (HMF), and their metabolites, influence metabolic parameters in mice with pre‐existing obesity. Differences in the effects of HMF and TAN may be attributed to the very different profiles of metabolites of these two compounds. Unlike TAN, HMF had dramatic influence in alleviating liver steatosis in obese mice.

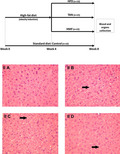

## 1 INTRODUCTION

A diet rich in saturated fats and high in calories is associated with cardiometabolic risk factors, including abdominal obesity, dyslipidemia, hyperglycemia, and high blood pressure. These factors also contribute to the occurrence of metabolic syndrome and type 2 diabetes (Grundy et al., 2005), which arise in part from adipose tissue inflammation and oxidative stress (Fernández‐Sánchez et al., 2011; Fonseca‐Alaniz et al., 2007; Kuryszko et al., 2016). In contrast, healthy diets rich in fruits and vegetables are associated with a normalization of inflammation and sharp drops in oxidant levels (Cassidy et al., 2012; Eichelmann et al., 2016; Jannasch et al., 2017; Rathee et al., 2009). These effects have been attributed to bioactive compounds in fruits and vegetables, and for citrus, these beneficial compounds largely include two main classes of flavonoids: the flavanone glycosides (i.e., hesperidin and naringin) and the more lipophilic polymethoxylated flavones (PMFs). Tangeretin (4',5,6,7,8‐pentamethoxyflavone; TAN) and heptamethoxyflavone (3',4',3,5,6,7,8‐heptamethoxyflavone; HMF) are among several of the PMFs that have been widely studied for their anti‐inflammatory and antioxidant properties (Arab et al., 2016; Assini et al., 2013; Kou et al., 2018; Lai et al., 2013; Lee et al., 2010, 2016; Liu et al., 2019; Manthey & Bendele, 2008). Inflammation and oxidative damage driven by stimulated macrophages in adipose tissue are major contributors to the development of metabolic syndrome and diabetes, and due to this, inhibition of macrophage‐driven inflammation is an important target in the discovery of compounds capable of combatting obesity‐associated diseases (Itoh et al., 2011). A recent study showed that TAN reduces cell damaging oxidative stress in STZ‐induced INS‐1 cells (Liu et al., 2019). Diabetic glucose loading induces chronic hypoxia, a mechanism commonly leading to diabetic nephropathy. TAN has been recently shown to inhibit diabetic glucose‐mediated hypoxia and the associated oxidative stress‐induced fibrotic injury (Kang et al., 2020). Diabetic obese mice treated with TAN at doses of 200 mg/kg experience reductions in body weight, insulin resistance and lowered secretion of key inflammation markers, adiponectin, leptin, resistin, IL‐6, and MCP‐1. (Kim et al., 2012) TAN ingestion mediated activities of enzymes important for carbohydrate metabolism and reduced glycosylated hemoglobin in diabetic rats (Sundaram et al., 2014). HMF has been also reported to regulate body weight, lipid profiles, and blood serum lipid levels in rats continuously fed a high‐fat diet (HFD; Feng et al., 2019). Nobiletin (3′,4′,5,6,7,8‐hexamethoxyflavone), a structurally related polymethoxylated flavone (PMF), improves adiposity, dyslipidemia, hyperglycemia, and insulin resistance in obese mice and also regulates glucose transport in muscles and white adipose tissue (Lee et al., 2010, 2013). This is in agreement with studies showing that TAN and nobiletin increase glucose uptake in murine adipocytes (Onda et al., 2013), and cocultures of hypertrophic adipocytes and macrophages (Shin et al., 2017).

The goals of this investigation were to evaluate the degrees of normalization of diabetes‐related indicators in mice with previously established obesity and to measure these main clinical signs of metabolic syndrome after administration of HFDs supplemented with TAN or HMF. Studies were done to characterize the mitigation of oxidative damage by both TAN and HMF, as well as to investigate the possible normalization of the blood glucose, insulin, cytokines, and adipose‐derived adipokines. Determinations were also made of differences in the effects of TAN and HMF on the above metabolic parameters. It is proposed that differences in the biological effects of HMF and TAN arise from the wide variations in the numbers and structures of circulating metabolites in mice receiving these two different PMFs. HPLC‐MS analysis of the metabolites of TAN and HMF in urine of Wistar rats was an initial approach to this study.

## 2 MATERIALS AND METHODS

### 2.1 Chemicals

Thiobarbituric acid, malondialdehyde, Trolox, and ABTS were purchased from Sigma‐Aldrich. Commercial sources for in vitro biological assay kits are listed in the following subsections. TAN and 3′,4′,3,5,6,7,8‐heptamethoxyflavone were purified from nonvolatile residues of orange peel oil (Gonçalves et al., 2018). Blood serum lipid analysis kits were purchased from Labtest (MG Brazil). Pure authentic standards of TAN and heptamethoxyflavone were obtained from the ARS citrus flavonoid collection (U.S. Horticultural Research Laboratory).

### 2.2 Animals and dietary treatment

**FIGURE 1:**
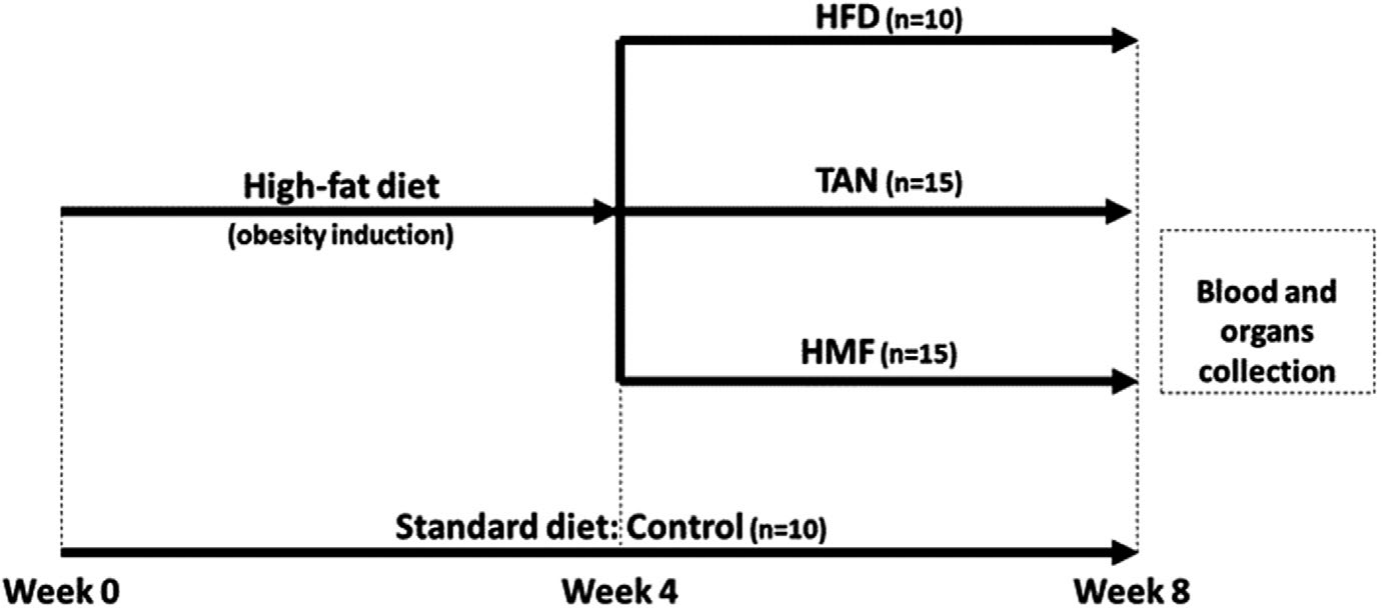
Experimental design. To obesity induction, high‐fat diet was given for 4 weeks. After that, HFD group continued to receive only high‐fat diet, and TAN and HMF groups received high‐fat diet supplemented with tangeretin and heptamethoxyflavone (100 mg/kg), respectively. Control group received standard diet during eight consecutive weeks

Six‐week‐old male C57BL/6J mice (São Paulo University, Ribeirão Preto, SP, Brazil) were maintained in an isolated system at 22 ± 2°C with a 12 hr light/12 hr dark cycle and free access to food and water. The experimental procedures were approved by the Ethics Committee on the Use of Animals of the School of Pharmaceutical Sciences, UNESP, Araraquara, SP, Brazil (Protocol CEUA/FCF/CAr no. 54/2015). After 1 week of adaptation, the mice were randomly divided into four groups with similar body weight distributions, including a control group fed a standard diet (C, *n* = 10); a HFD (*n* = 10); a HFD supplemented with TAN at a dose of 100 mg/kg body weight (TAN, *n* = 15); and a HFD supplemented with HMF at a dose of 100 mg/kg body weight (HMF, *n* = 15) in a manner similar to that described by Ferreira et al. (2016). The time course of the feeding trial is shown in Figure 1. The purified TAN and HMF were mixed separately into the diet for each animal, based on the food intake of the previous day (grams of food ingested per day) with an additional of 10% to ensure the intake of the daily dose. The food intake was monitored at regular intervals of 24 hr, and the supplements’ amounts were adjusted accordingly. Body weights of the mice were monitored weekly and the food intake was monitored daily, always at the same time of the day. The compositions of the standard diet (11% lipids) and the HFD (45% lipids) are shown in Table 1. The experimental design was developed to observe the effects of TAN and HMF on mice with pre‐existing obesity. At the end of the 8th week, mice were anesthetized by xylazine/ketamine (16/60 mg/g of body weight) i.p. injection and euthanized by cardiac puncture. Blood serum was obtained by centrifugation, and the organs were stored at −80°C.

**TABLE 1:** Composition of standard and high‐fat diet (manufactured with pure ingredients by Rhoster Industry and Trade LTD, SP, Brazil)

Diet	Standard	High‐fat
Energy (kcal/g)	4.27	5.35
Protein (% kcal)	14.6	20.8
Carbohydrates (% kcal)	73.9	33.8
Lipids (% kcal)	11.5	45.3
**Composition**	**g/100 g**	
Corn starch	46.6	7.8
Casein	14.0	24.0
L‐Cisteine	0.2	0.4
Maltodextrin	15.5	11.7
Sucrose	10.0	20.1
Fiber	4.0	5.8
Soybean oil	5.0	2.9
Lard	‐	20.7
Mineral mix	3.5	5.2
Vitamin mix	1.0	1.2
Choline bitartrate	0.3	0.2
Total	100.0	100.0

TAN and HMF were isolated from crude residues remaining after high‐vacuum distillation of cold‐pressed orange peel oil (Gonçalves et al., ). Final steps of TAN and HMF purification involved crystallization from acetone at −20°C, followed by washing the recovered compounds with ice‐chilled hexane. The final purities of the TAN and HMF were >95% and >98%, respectively, compared with authentic standards.

### 2.3 Blood serum analyses

Fasting (12 hr) levels of blood serum glucose, triglycerides, total cholesterol (total‐C), HDL cholesterol (HDL‐C), alanine transaminase (ALT), and aspartate transaminase (AST) were evaluated by enzymatic colorimetric assays using commercial kits (Labtest). Non‐HDL‐C was calculated by the difference between total‐C and HDL‐C. The levels of insulin and the cytokines: TNF‐α, MCP‐1, IL‐10, and the adipokines: adiponectin, resistin, and leptin were determined by Multiplex Luminex XMAP detection method (Merck KGaA).

### 2.4 Oxidative stress parameters

Blood serum oxidative stress was measured by lipid peroxidation using the thiobarbituric acid‐reactive substances (TBARS) assay and quantified in μM malondialdehyde (Yagi, 1998). Total antioxidant capacity in blood serum was evaluated by the ABTS assay (Janaszewska & Bartosz, 2002). The absorbance was measured at 734 nm to verify the formation of ABTS●+ and, to prepare the calibration curve, Trolox (Sigma) was used as a standard. The antioxidant capacity was determined as mM of Trolox equivalent antioxidant capacity (TEAC). All blood serum oxidative stress and total antioxidant capacity analyses were performed in triplicate.

### 2.5 Organs histology

Immediately after euthanasia, blocks of intra‐abdominal adipose tissue and the left lobe of liver were carefully dissected from the animals, rinsed in saline 0.9%, fixed in buffered formalin for 48 hr, and kept in 80% ethanol. Tissues were submitted to routine processing for paraffin embedding, sectioned to 4–6 µm thickness, and stained with hematoxylin and eosin. Histological images were obtained using a digital camera on an optical microscope under 100x magnification, and areas of ≥40 adipocytes were measured in each photo, using computerized software (UTHSCSA ImageTool, version 3.0). A pathologist evaluated the anonymized histological samples by optical microscopy to identify morphologic alterations between groups.

### 2.6 Isolation of TAN and HMF metabolites from rat urine

A separate feeding trial was performed to identify metabolites of TAN and HMF. Twenty male Wistar rats weighing 180 ± 5 g were separated into two groups, and placed in individual metabolic cages and maintained under controlled conditions of 23 ± 1°C room temperature, 55 ± 5% relative humidity, and a 12–12 hr day–night cycle. Each group, consisting of 10 animals each, was fed a normal laboratory chow diet (Purina Evialis do Brasil Nutrição Animal Ltda.) with free access to food and water. After a week of acclimation, the rats received TAN or HMF at a dose of 100 mg/kg body weight, mixed into plain yogurt, at a volume of 1.0 ml/day, and given daily by gavage for 15 days (Assis et al., 2017; Gutierres et al., 2015; Manach et al., 2005), with free access to food and water. For each of the 15 days, urine from each rat was collected at 8 a.m. and 5 p.m., and each entire daily volume of urine was stored at −80°C. The combined rat urine from the 15 days of treatments was added in 200 ml increments to 400 ml 0.05 M aqueous formic acid and subsequently extracted into ethyl acetate. Extractions were performed in triplicate. The combined ethyl acetate phases were evaporated to near dryness with a rotary evaporator. HPLC‐MS analysis of the metabolites before and after ethyl acetate extraction and vacuum drying showed no evidence of hydrolysis. Subsequent metabolite isolations were achieved by silica gel column chromatography and preparative‐scale C18 reversed phase HPLC as reported previously (Gonçalves et al., 2018; Manthey et al., 2011). Analyses of the TAN and HMF metabolites were performed with a Waters 2695 Alliance HPLC (Waters) connected in parallel with a Waters 996 PDA detector and a Waters/Micromass ZQ single‐quadrupole mass spectrometer equipped with an electrospray‐ionization (ESI) source. Compound isolations and HPLC‐ESI‐MS detections and analyses were achieved as previously described (Gonçalves et al., ; Manthey et al., 2011), and preliminary structural identifications are presented in subsequent sections. Substitution patterns of metabolite flavone A‐ and B‐rings were analyzed by the fragmentation ions formed by retro‐Diels − Alder ring fissions created by HPLC‐ESI‐MS operated at elevated cone voltages (Berahia et al., 1994; Chen et al., 1997; Rizzi & Boeing, 1984).

### 2.7 Statistical analysis

All results were expressed as mean ± *SD*. The normality of the data was tested, and the variation between the groups was measured by one‐way analysis of variance (ANOVA) followed by post hoc analysis (Tukey test), to evaluate the effects caused by the HFD consumption and/or the supplementation with TAN or HMF, with significance level *p* < .05 (Sigma Stat Software).

## 3 RESULTS

### 3.1 Effects of TAN and HMF on dietary intake, body weight gain, and organs

**FIGURE 2:**
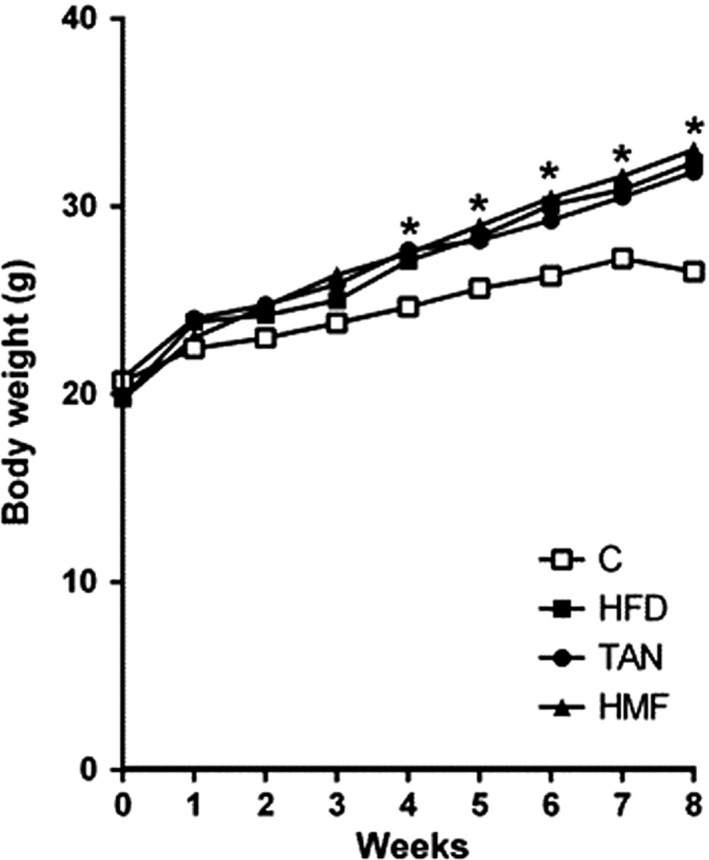
Body weights of male C57BL/6J mice fed with standard diet (C), high‐fat diet (HFD), or HFD supplemented with tangeretin (TAN) or heptamethoxyflavone (HMF). *p < .05 for HFD, TAN, and HMF groups in comparison with the control

The HFD, TAN, and HMF groups received identical diets up to the fourth week, and as shown in Figure 2, these three groups showed identical growth rates and were not statistically different from the normal control diet group during this period. A previous study using identical experimental conditions confirmed the full development of obesity in mice by the fourth week (Ferreira et al., 2016). Between weeks 4 and 8, the growth in the three HFD groups increased over the control group's weight gain (*p* < .05), but the addition of TAN and HMF to these diets during this time period produced no decreases in weight gains relative to the nonsupplemented HFD group (Figure 2). In a recent study, differences between weight gains in rats receiving either a nonsupplemented HFD or a HFD supplemented with HMF were not statistically different until after 4 weeks (Feng et al., 2019), and the results in Figure 2 are in agreement with this. As shown in Table 2 the HFD, TAN and HMF obese groups had lower dietary intakes, but showed heavier weights of intra‐abdominal adipose tissue compared with control group (*p* < .05). Animals in the TAN and HMF groups exhibited heavier kidneys (6%) compared with the control group (*p* < .05), but there were no differences in the weights of the livers, spleen, and hearts among the control and three HFD groups. In contrast to this, the HFD produced a 31% increase in mouse pancreas weights, but TAN and HMF supplementation blocked this HFD‐induced pancreas weight increase (*p* < .05).

**TABLE 2:** Organ weights and adipocyte size of male C57BL/6J mice fed with standard diet (Control), high‐fat diet (HFD), or high‐fat diet supplemented with tangeretin (TAN) or heptamethoxyflavone (HMF)

Variables	Control	HFD	TAN	HMF
Energy intake (kcal)	15.4 ± 0.4^b^	14.0 ± 1.1^a^	14.5 ± 1.0^a^	14.2 ± 0.7^a^
Liver (g)	1.24 ± 0.09	1.19 ± 0.17	1.21 ± 0.12	1.18 ± 0.09
Kidney (g)	0.34 ± 0.01^a^	0.36 ± 0.03^a^	0.38 ± 0.03^b^	0.38 ± 0.04^b^
Pancreas (g)	0.16 ± 0.02^a^	0.21 ± 0.05^b^	0.16 ± 0.03^a^	0.17 ± 0.02^a^
Spleen (g)	0.08 ± 0.01	0.09 ± 0.02	0.08 ± 0.01	0.09 ± 0.01
Heart (g)	0.13 ± 0.01	0.13 ± 0.01	0.15 ± 0.01	0.14 ± 0.01
Adipose tissue^a^ (g)	0.38 ± 0.08^a^	2.11 ± 1.05^b^	1.48 ± 0.73^b^	1.87 ± 0.66^b^
Adipocyte area (µm^2^)	13.6 ± 1.6^a^	45.0 ± 7.1^b^	37.05 ± 7.5^a^	46.5 ± 6.4^b^

Note

Results are presented as mean ± *SD*. Data analyzed by 1‐factor ANOVA, followed by Tukey's test. Mean in a row followed by different letters differ significantly (*p* ≤ .05).

^a^ Intra‐abdominal adipose tissue.

### 3.2 Histopathological evaluation of adipocytes and hepatic tissue

**FIGURE 3:**
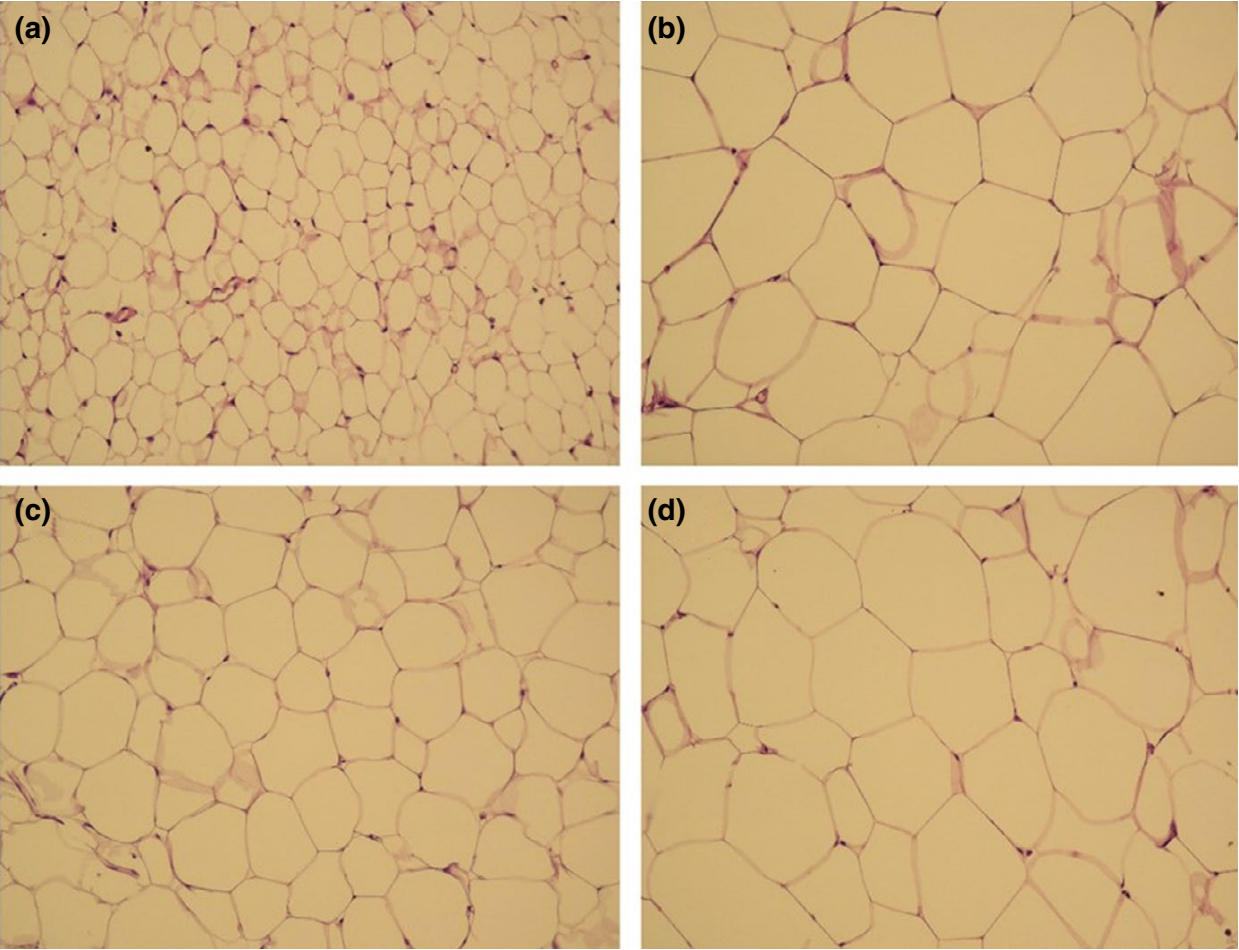
Histological sections of intra‐abdominal adipose tissue of mice fed with standard control diet (a), HFD (b), HFD diet supplemented with TAN (c), or HMF (d; 100× magnification)

**FIGURE 4:**
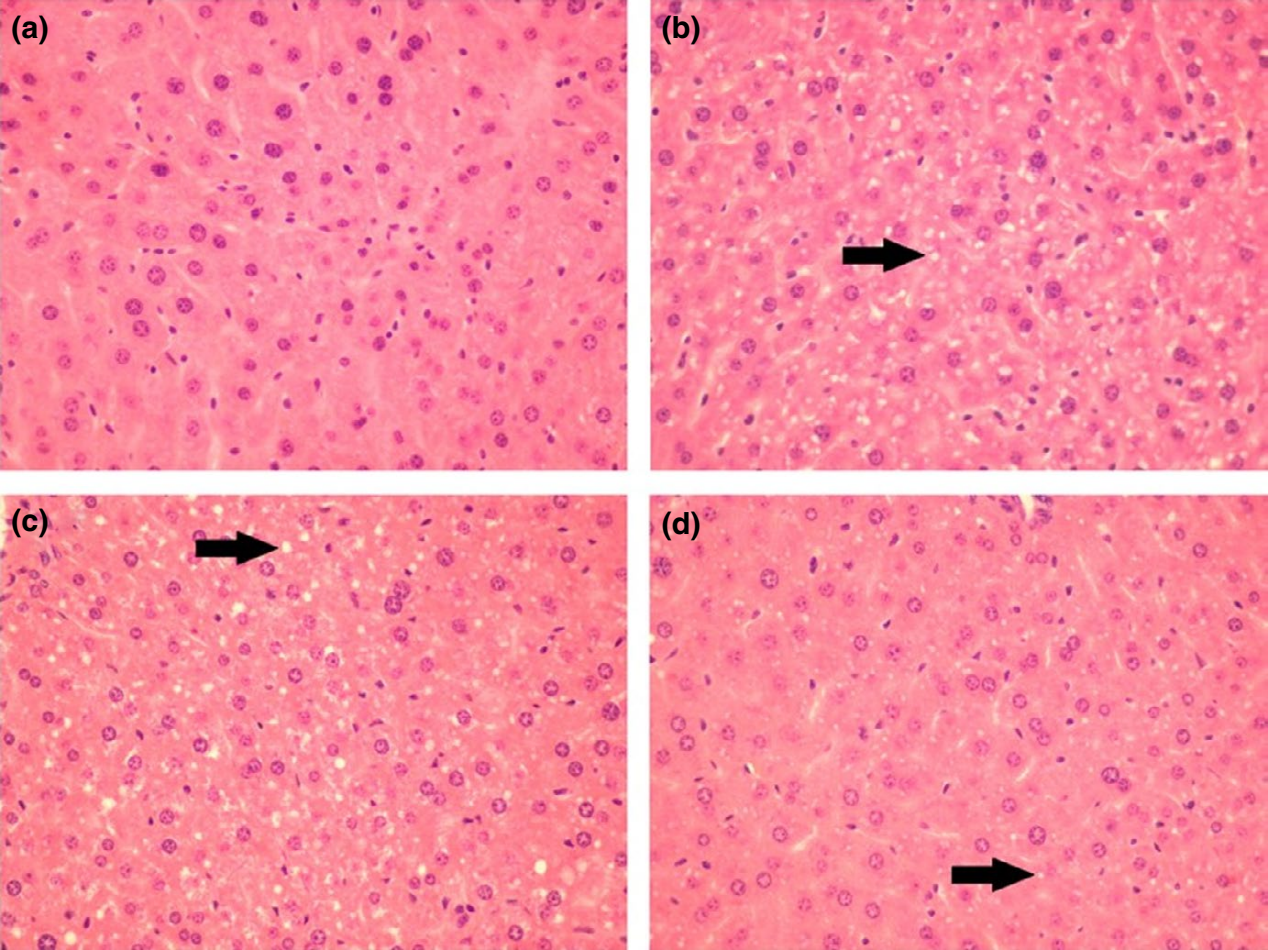
Histological sections of liver tissue of mice fed with standard diet (a), HFD (b), HFD supplemented with TAN (c), or HMF (d; 400× magnification). Arrows in b, c, d point to characteristic fat vacuoles that occur with liver steatosis and appear colorless with the hematoxylin and eosin and Masson's trichromic stains

Histopathological evaluations were used to characterize the effects of the HFD and supplements on adipose and liver tissues in the obese C57BL/6J mice. In the adipose tissue, the nonsupplemented HFD and the HMF‐supplemented HFD groups showed larger adipocytes in comparison with control (*p* < .05; Table 2 and Figure 3). As represented in Figure 3c, the adipocytes in the TAN‐supplemented group tended to appear smaller than those of the HFD and HMF groups (Figure 3b,d, respectively), although measurements of mean cross areas showed no statistical differences (*p* < .05; Table 2). In the liver, the HFD and TAN groups exhibited granular cytoplasm and diffused distributions of macro vesicular fat deposits and were diagnosed with liver steatosis (Figure 4b,c, respectively). In contrast, the HMF group showed smaller fat deposits and less pronounced liver steatosis compared with the HFD and TAN groups (Figure 4d). The control group exhibited typical morphology, with normal microvesicular fat deposits (Figure 4a).

### 3.3 Biochemical profile in blood serum and liver

The elevated fasting blood glucose levels (241 ± 20 mg/dl) measured for the control group (Table 3) are typical in healthy mice following anesthesia with xylazine‐ketamine and were not indicative of preanesthesia hyperglycemia (Braslau et al., 2007; Ferreira et al., 2016). The insulin resistance (HOMA‐IR index) and blood glucose levels of the control group were significantly lower than for the HFD group (*p* < .05). No differences occurred in the insulin levels between the HFD, TAN, and HMF groups. The final blood glucose levels and insulin resistance for the TAN and HMF groups tended to occur between the HFD and control groups, but no statistical difference was observed (*p* < .05). Non‐HDL‐C was higher in the HFD, TAN, and HMF groups compared with the control group (*p* < .05). Blood serum triglycerides, total‐C, HDL‐C, ALT, and AST showed no significant differences among the four experimental groups (Table 3). Liver triglyceride levels in the HFD, TAN, and HMF groups were significantly higher than in the control group (*p* < .05), while liver cholesterol was not different among any of the groups (Table 3). No significant differences in the blood serum triglyceride levels were measured for the four experimental groups.

**TABLE 3:** Biochemical profile and liver lipids of male C57BL/6J mice fed with standard diet (Control), high‐fat diet (HFD), or high‐fat diet supplemented with tangeretin (TAN) or heptamethoxyflavone (HMF)

Variables	Control	HFD	TAN	HMF
Biochemical profile				
Glucose (mg/dl)	241 ± 20^a^	343 ± 63^b^	285 ± 78^ab^	317 ± 36^ab^
Insulin (pg/ml)	74 ± 16	130 ± 61	129 ± 51	109 ± 19
HOMA‐IR	1.27 ± 0.27^a^	2.73 ± 1.19^b^	2.43 ± 1.06^ab^	2.45 ± 0.47^ab^
Triglycerides (mg/dl)	75 ± 6	71 ± 13	76 ± 10	76 ± 11
Total cholesterol (mg/dl)	88 ± 7	100 ± 10	101 ± 15	107 ± 16
HDL cholesterol (mg/dl)	50 ± 7	50 ± 5	53 ± 10	55 ± 6
Non‐HDL chol. (mg/dl)	38 ± 5^a^	49 ± 6^b^	48 ± 7^ab^	49 ± 7^b^
ALT (mg/dl)	49 ± 20	69 ± 20	66 ± 27	46 ± 19
AST (mg/dl)	279 ± 121	326 ± 125	256 ± 92	247 ± 76
Liver lipids				
Triacylglycerol (mg/g)	114 ± 25^a^	217 ± 62^b^	204 ± 40^b^	219 ± 55^b^
Cholesterol (mg/g)	20 ± 1.4	22 ± 4.2	23 ± 2.5	21 ± 1.8

Note

Results are presented as mean ± *SD*. Data analyzed by 1‐factor ANOVA, followed by Tukey's test. Mean in a row followed by different letters differ significantly (*p* ≤ .05). Only statistical differences are described with letters.

### 3.4 Inflammation and oxidative stress biomarkers

Blood serum leptin and resistin were significantly higher in the HFD group compared with the control group. The mean leptin and resistin levels for the TAN and HMF groups occurred between the mean values for the HFD and control groups, but no statistical difference was observed (*p* < .05). Adiponectin levels were similar in the four experimental groups (Table 4). IL‐10 levels were not different between the control, HFD, and TAN groups, but were much higher in the HMF group (*p* < .10). MCP‐1 and TNF‐α did not differ between the groups (Table 4). Blood serum lipid peroxidation value (TBARS) of the HFD group was significantly higher than the control group. This large increase was nearly completely blocked by TAN and HMF supplementation (*p* < .05). Serum antioxidant capacities (ABTS assay) were not altered by the HFD, supplemented, or not (Table 4).

**TABLE 4:** Inflammatory and oxidative stress biomarkers of male C57BL/6J mice fed with standard diet (Control), high‐fat diet (HFD), or high‐fat diet supplemented with tangeretin (TAN) or heptamethoxyflavone (HMF)

Variables	Control	HFD	TAN	HMF
Adiponectin (mg/L)	3.20 ± 0.54	3.18 ± 0.84	2.77 ± 0.66	3.58 ± 0.78
Leptin (pg/ml)	353 ± 150^a^	10,247 ± 6,477^b^	4,757 ± 2,287^ab^	6,094 ± 3,315^ab^
Resistin (pg/ml)	1,479 ± 324^a^	2,201 ± 136^b^	1,707 ± 383^ab^	1,730 ± 387^ab^
MCP−1 (pg/ml)	37.7 ± 8.0	36.6 ± 6.4	38.8 ± 6.4	38.6 ± 10.3
TNF‐α (pg/ml)	2.79 ± 0.45	3.16 ± 0.67	3.32 ± 0.58	3.26 ± 0.67
IL−10 (pg/ml)	2.02 ± 0.98	1.65 ± 0.73	2.01 ± 0.69	3.37 ± 1.55*
TBARS (μM)	6.43 ± 0.87^a^	12.55 ± 3.06^b^	7.37 ± 1.27^a^	8.75 ± 1.56^a^
ABTS (mMeq Trolox)	1.40 ± 0.04	1.41 ± 0.06	1.38 ± 0.06	1.44 ± 0.03

Note

Results are presented as mean ± *SD*. Data analyzed by 1‐factor ANOVA, followed by Tukey's test. Mean in a row followed by different letters differ significantly (*p* ≤ .05). Only statistical differences are described with letters.

* *p*‐value is significantly different (*p* < .10) comparing HFD and HMF groups.

### 3.5 TAN and HMF metabolites

**FIGURE 5:**
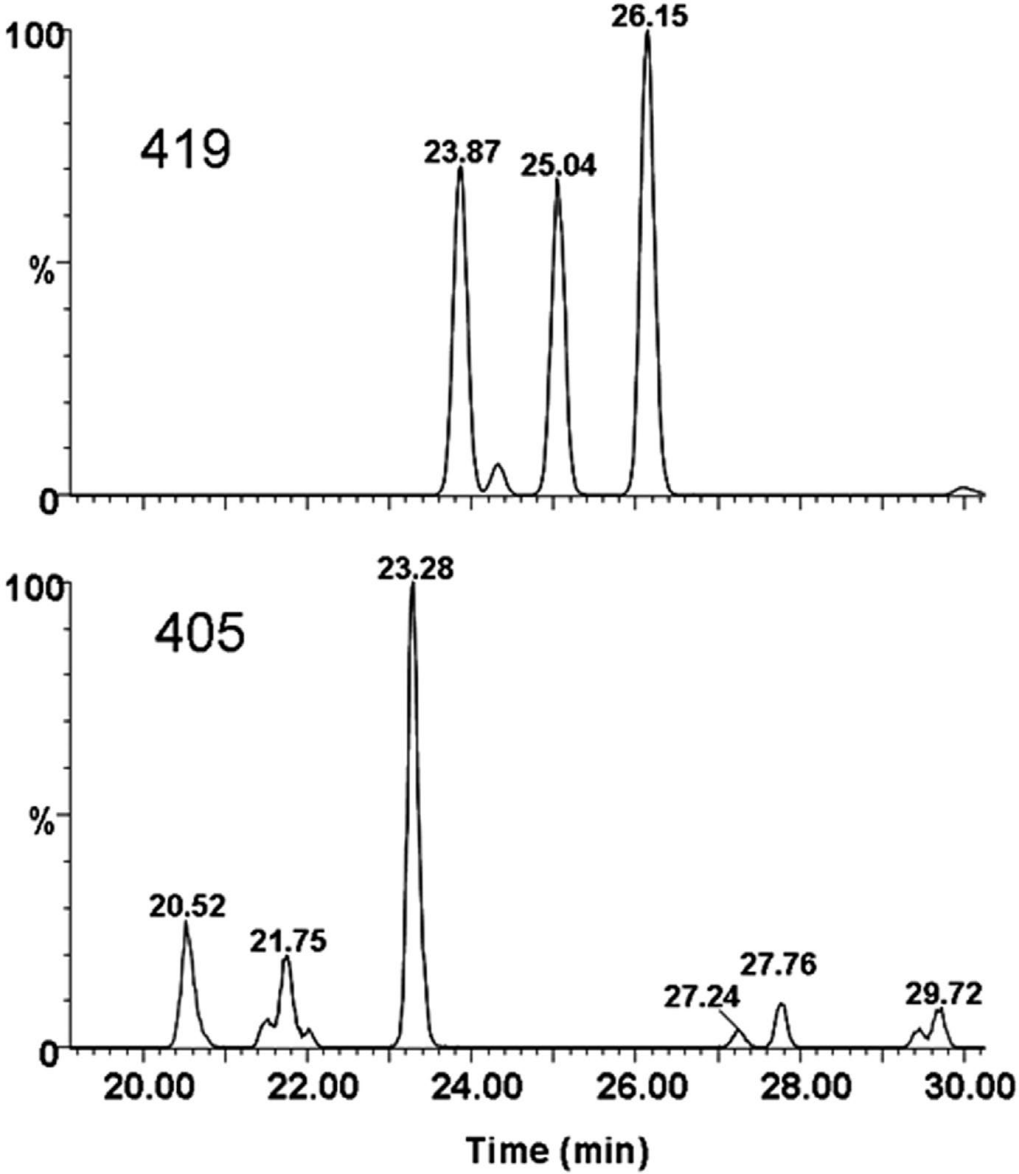
ESI‐MS chromatograms with peak extraction at 419 *m/z* (top) and 405 *m/z* (bottom)

**FIGURE 6:**
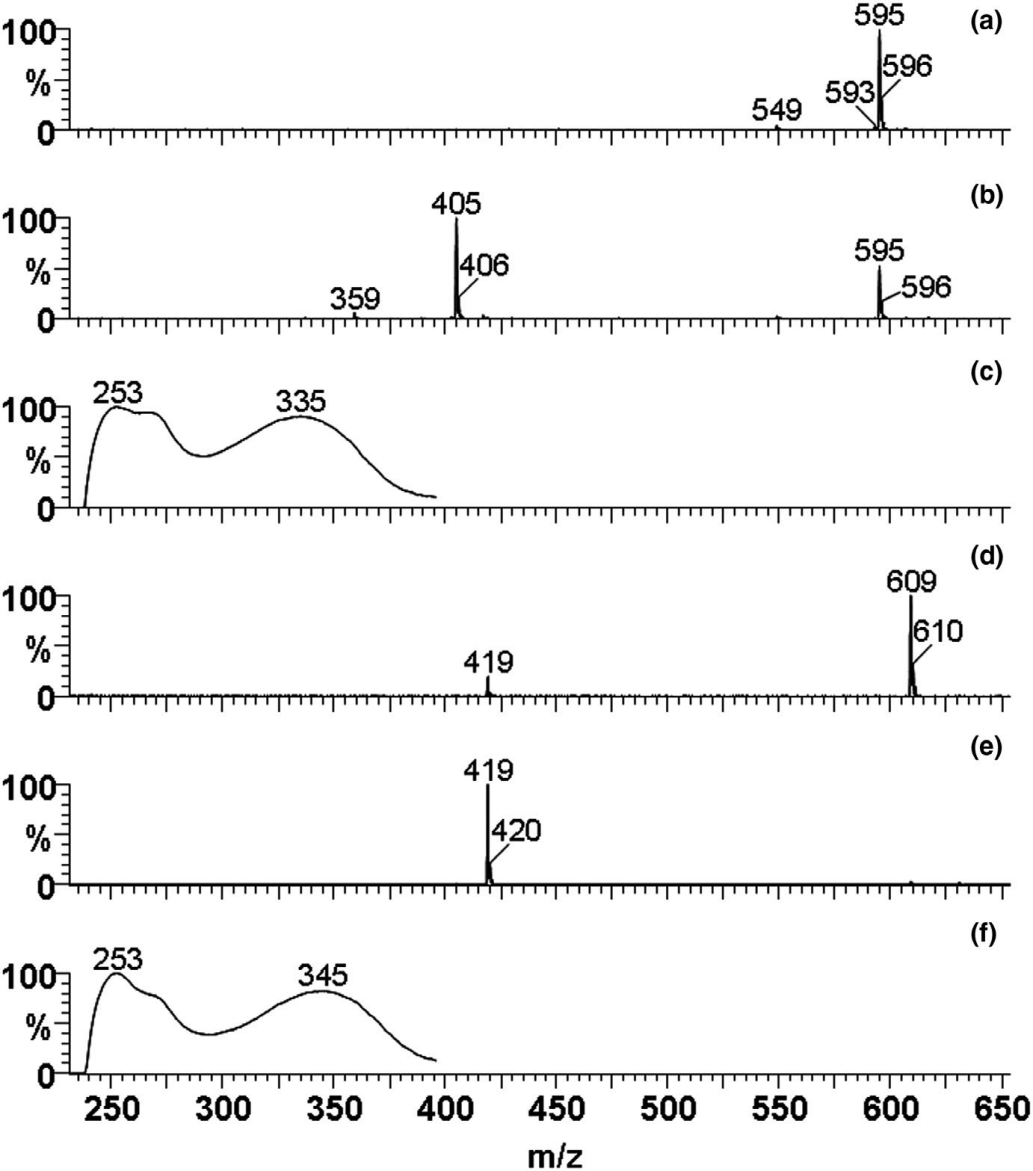
ESI‐MS of representative metabolites with 190 amu neutral losses indicative of cleavage of methylglucuronate substituents of HMF metabolites at 21.9 and 22.6 min. Figures a and d show low fragmentation energy mass spectra and the intact protonated molecular ions *m/z* 595 and 609, respectively, while Figures b and e show higher fragmentation energy mass spectra, illustrating the protonated mass ions of the di‐desmethylHMF and mono‐desmethylHMF fragments of *m/z* 405 and 419, respectively. Figures c and f report the UV spectra of the two HMF‐methylglucuronate metabolites

Wistar rats were used to facilitate the detection of TAN and HMF metabolites and thus gain an understanding of the scope of chemical structures potentially involved in this study. The HPLC‐ESI‐MS analysis of the urine of Wistar rats, dosed with TAN, showed only two main metabolites: 4′‐desmethylTAN (i.e., 4′‐hydroxy‐5,6,7,8‐tetramethoxyflavone) and TAN‐4′‐*O*‐glucuronide (i.e., 5,6,7,8‐tetramethoxyflavone‐4′‐*O*‐glucuronide; Table 5). This observation is similar to those of earlier studies (Breinholt et al., 2003; Cheng et al., 2011; Manthey et al., 2011; Nielsen et al., 2000). These two compounds were identified by their monodesmethylTAN protonated mass ions (M + H)^+^ at *m/z* 359, and at both *m/z* 535 and 359 for the monodesmethylTAN glucuronide (Kurowska & Manthey, 2004; Manthey et al., 2011). Both compounds showed exact peak overlaps with established standards of 4′‐desmethylTAN and TAN‐4′‐*O*‐glucuronide (4′‐hydroxy‐5,6,7,8‐tetramethoxyflavone‐4′‐*O*‐glucuronide; data not shown). In contrast to this, the same measurement techniques enabled the detection of 18 HMF metabolites (Table 6). The monodesmethylated and didesmethylated metabolites of HMF were detected at *m/z* 419 and 405, respectively, along with their simultaneous detection of *m/z* 595 and 581 ions, respectively, for the monodesmethyl and di‐desmethylHMF glucuronides. Identifications of these metabolites were further confirmed by the detection of the MS‐induced retro‐Diels‐Alder fragmentation ion profiles characteristic of substituted flavones. The results in Table 6 show the degrees of desmethylation of the flavone A‐ and B‐rings (Berahia et al., 1994; Chen et al., 1997; Rizzi & Boeing, 1984). Three di‐desmethylHMF aglycones (A‐C) and three mono‐desmethylHMF aglycones (D‐F) were detected (Figure 5), and later isolated for preliminary structural analysis. The mass spectrum of C showed fragment ions at *m/z* 211/183/137/135, consistent with a tetramethoxy A‐ring and a B‐ring with a 3′,4′‐dihydroxy phenyl structure. Three aglycone metabolites (A,B,D) exhibited A‐ring mono‐desmethylation by the occurrence of fragment ions at *m/z* 197 and 169. Five mono‐desmethylHMF‐*O*‐glucuronides (H,I,K,M,N) and three di‐desmethylHMF‐*O*‐glucuronides (G,J,L) were detected. Each of these showed neutral losses of 176 amu (presumed glucuronic acid‐H_2_O) in their positive ion ESI‐MS. Four metabolites (O‐R) exhibited neutral losses of 190 amu (Figure 6), which are tentatively assigned to the losses of methylglucuronide (Hammoud et al., 2012; Nazaruk & Jakoniuk, 2005; Rodriguez Lanzi et al., 2018; Serra et al., 2009).

**TABLE 5:** TAN metabolites, protonated molecular weights, chromatographic elution times, and flavone A‐ and B‐ring substitution patterns

*m/z*	Elution time (min)	Structure by ESI‐MS fragmentation
535/359	18.6	Monohydroxy A‐ring; tetramethoxy B‐ring + glucoronide^a^
359	25.2	Monohydroxy A‐ring; tetramethoxy B‐ring^b^

^a^ 4‐hydroxy‐5,6,7,8‐tetramethoxyflavone‐4′‐*O*‐glucuronide).

^b^ 4′‐hydroxy‐5,6,7,8‐tetramethoxyflavone).

**TABLE 6:** HMF metabolites, protonated molecular weights, chromatographic elution times, and flavone A‐ and B‐ring substitution patterns

[M + H]^+^	Elution time (min)	Structure by ESI‐MS fragmentation
Aglycone		
A 405	20.6	trimethoxy‐monohydroxy A‐ring; monomethoxy‐monohydroxy B‐ring
B 405	21.7	trimethoxy‐monohydroxy A‐ring; monomethoxy‐monohydroxy B‐ring
C 405	23.3	3′,4′‐dihydroxy−3,5,6,7,8‐pentamethoxyflavone
D 419	23.9	trimethoxy‐monohydroxy A‐ring; dimethoxy B‐ring
E 419	25.1	tetramethoxy A‐ring; monomethoxy‐monohydroxy B‐ring
F 419	26.2	tetramethoxy A‐ring; monomethoxy‐monohydroxy B‐ring
Glucuronide		
G 581/405	16.2	di‐desmethylHMF‐*O*‐glucuronide
H 595/419	17.1	mono‐desmethylHMF‐*O*‐glucuronide
I 595/419	18.3	mono‐desmethylHMF‐*O*‐glucuronide
J 581/405	18.6	di‐desmethylHMF‐*O*‐glucuronide
K 595/419	19.9	mono‐desmethylHMF‐*O*‐glucuronide
L 581/405	20.3	di‐desmethylHMF‐*O*‐glucuronide
M 595/419	20.3	mono‐desmethylHMF‐*O*‐glucuronide
N 595/419	20.9	mono‐desmethylHMF‐*O*‐glucuronide
O 609/419	21.3	mono‐desmethylHMF‐*O*‐methylglucuronide
P 595/405	21.9	di‐desmethylHMF‐*O*‐methylglucuronide
Q 609/419	22.6	mono‐desmethylHMF‐*O*‐methylglucuronide
R 609/419	23.2	mono‐desmethylHMF‐*O*‐methylglucuronide

## 4 DISCUSSION

Rapid advancements have been made in understanding the underlying biochemical modes of action of PMFs in combatting diabetes and obesity. New pathways may occur via the influences of these compounds on gut microbiota and their production of numerous diverse metabolites (Chen et al., 2020). In this current study, the effects of TAN and HMF added to a HFD were investigated in C57BL‐6J mice with pre‐existing obesity. Results of this investigation showed modest improvements by TAN and HMF in metabolic parameters in obese mice, where the mean values for fasting blood glucose, insulin resistance, blood serum resistin, and leptin tended to occur between the values for the control healthy diet animals and the nonsupplemented HFD group, although no statistical differences occurred among the HFD groups. Significant reductions in blood serum lipid peroxidation did occur as a result of TAN and HMF inclusion in the HFD. Several novel findings in this study included significantly higher levels of the anti‐inflammatory cytokine IL‐10 in the HMF group. Both TAN and HMF prevented the significantly increased pancreas weights observed in the mice on the nonsupplemented HFD. This suggests a possible protection against HFD‐linked pancreatic inflammation (Esser et al., 2014) by these citrus compounds. Histological analyses of the adipocytes and liver tissues showed that TAN, but not HMF, tended to reduce the size of adipocytes, similar to effects previously reported by Kim et al. (2012). In the liver tissue, only HMF, but not TAN, decreased steatosis in the obese mice. Results such as these provide initial indications of possible differences between the effects of TAN and HMF in obese mice.

In this study, no effects of TAN and HMF were observed on the blood serum or liver lipid profiles in the obese mice. These findings are different from those of a recent investigation where TAN exhibited significant antilipogenic and other lipid‐lowering effects in HFD‐fed rats. TAN ingested as 0.04, and 0.08 percent of the HFD for 6 weeks significantly lowered body weight gains. The lower body weights were mainly attributable to decreased adipose tissue produced with supplementation with TAN (Feng et al., 2020). In these HFD‐fed rats, the antiobesity and cholesterol‐lowering effects of TAN were linked to the ability of TAN to modulate hepatic lipid biosynthesis, particularly shown by sharply lowered levels of serum triglycerides and total‐C. Similar findings were made with hamsters fed diets rich in TAN and nobiletin (Kurowska & Manthey, 2004).

The antidiabetic properties of TAN were earlier studied in mice administered daily at doses of 200 mg/kg in a HFD for 8 weeks (Kim et al., 2012), but with no predose obesity development. After 8 weeks, the dosed mice on the HFD exhibited significantly lower weight gain, glucose tolerance, total‐C levels, and decreased secretion of proinflammatory adipocytokines and cytokines compared with the nonsupplemented HFD mice. Wild‐type C57BL/6J mice on a normal diet with intragastric dose**s** of TAN for 30 days produced significantly lower serum glucose levels and serum insulin levels in nonfasted animals on day 31 (Guo et al., 2020). Blood glucose tolerance test**s** after 16‐hr fasting showed significantly lower glucose levels for 25 and 50 mg/kg dosed animals compared with nontreated animals, but insulin resistance was significantly different only for the mice dosed with 50 mg/kg (Guo et al., 2020). In a further study, 30 days of oral administration of TAN (100 mg/kg body weight) to streptozotocin‐induced diabetic rats resulted in significant reductions in plasma glucose and glycosylated hemoglobin (HbA1c; Sundaram et al., 2014). The TAN‐treated streptozotocin‐induced diabetic rats showed regeneration of pancreatic islets compared with the diabetic nontreated control group. Improved glucose tolerance, reduced adipocytokine production, and inhibition of fat accumulation in mice fed a HFD have been additionally reported by Lee et al. (2016). In this study, TAN was also shown to inhibit LPS‐induced production of nitric oxide, TNF‐α, IL‐6, and IL‐1β. In our with TAN and HMF, neither compound had an effect on the production of cytokine production, although both compounds are potent inhibitors of IL‐6, IL‐1β, and TNF‐α production in human monocytes (Manthey et al., 1999) and were anticipated to act as such in the TAN‐ and HMF‐dosed animals. The reasons for not observing decrease the levels of inflammatory cytokines in the obese mice treated with HMF and TAN are uncertain, but they may be attributable to the pre‐establishment of adipose tissue in the obese mice prior to introductions of TAN and HMF to the final HFDs.

Lipid‐rich diets increase systemic oxidative stress by elevating the production of reactive oxygen species (ROS) and reducing the levels of the antioxidant superoxide dismutase, catalase, and glutathione peroxidase, all of which occur with an increased production of inflammatory cytokines (Fernández‐Sánchez et al., 2011; Fonseca‐Alaniz et al., 2007; Kuryszko et al., 2016). The presence of chronically higher ROS levels promotes apoptosis of pancreatic β‐cells, thus impairing insulin production. In the present study, both TAN and HMF were able to prevent the significant enlargement (31%) of the pancreas caused by the HFD consumption. Also, the supplementation with TAN and HMF decreased the lipid peroxidation (TBARS) in the blood serum, an oxidative stress parameter. For PMFs, the modulation of oxidative stress is due in part to the abilities of reduced inflammation by inhibiting the production of proinflammatory cytokines and ROS (Manthey et al., 1999; Nikaido et al., 1982; Rathee et al., 2009). Adipose tissue macrophages are major sources of proinflammatory cytokines, such as TNF‐α, IL‐6, and IL‐1ß, and the inhibition of adipose macrophage cytokine production by TAN and HMF, as well as by other PMFs, has been previously reported (Funaro et al., 2016; Shin et al., 2017). TAN has also been shown to enhance skeletal mitochondrial biogenesis by activating the AMPK‐PGC1‐a pathway which potentially inhibits the progression of metabolic syndrome and type‐2 diabetes in mammals (Kou et al., 2018). Our study provides evidence suggesting that HMF attenuates hepatic damage and steatosis caused by HFDs, with concurrent reductions in oxidative stress. A recent transcriptome analysis demonstrated that HMF supplementation markedly down‐regulated hepatic genes related to adipogenesis and inflammatory responses, while increasing fatty acid oxidation and energy expenditure (Feng et al., 2019), and we hypothesize that this can contribute to the observed effect of HMF on the steatosis mitigation.

Yet, chemical analyses of tissues of PMF‐dosed animals show that the overwhelming portions of PMFs occur as desmethylated aglycones ‐*O*‐glucuronides and sulfates. Because only trace amounts of the original PMFs are detected, it is commonly assumed that biological actions of the PMFs are largely due to their various metabolites. In fact, for several of the PMFs, desmethylated metabolites exhibit greater in vitro activities than the parent compounds (Cheng et al., 2011; Huang et al., 2016; Li et al., 2007, 2014; Sun et al., 2019; Wang et al., 2013, 2016; Zheng et al., 2013). In the present study, it was shown that are possibly twelve glucuronides among the HMF metabolites, while there is only one glucuronide among the main TAN metabolites. Another major difference among the HMF and TAN metabolites are the numerous di‐desmethylHMF aglycones and glucuronides. The chemical reactivities of a monohydroxy phenyl flavone B‐ring as occurring in the two main TAN metabolites (Table 5) are notably different from the reactivities of the didesmethyl and monodesmethyl adjacent substituents on the B‐ring phenyl group of HMF metabolites. Of particular, significance would be the catechol‐like 3′,4′‐dihydroxy substitution on the flavone B‐ring, as is seen for metabolite C in Table 6. Also, a series of methylglucuronate conjugates of HMF were detected in this study, and these compounds remain to be fully chemically characterized and tested in in vitro assays pertinent to the amelioration of diabetes and obesity. Yet, based on the recent findings of potent biological actions of numerous PMF metabolites, it is reasonable to surmise that the many different metabolites of HMF listed in Table 6 contribute in individual manners to the cumulative biological effects of HMF. It would be interesting to characterize whatever cellular sequestration and localization that may occur with the above TAN and HMF compounds in mammalian cells, as previously studied by Gonçalves et al. (2018).

## Data Availability

Data available on request from the authors.
